# Efficacy and Safety of Glycerol Lidocaine Ear Drops in the Non-Antibiotic Treatment of Otitis Externa Symptoms—An Observational Study

**DOI:** 10.3390/clinpract16050090

**Published:** 2026-04-30

**Authors:** Maria Sobol, Ewelina Sielska-Badurek, Mariusz Cięciara, Artur Wrzosek, Justyna Tomaszewska, Beata Roman

**Affiliations:** 1 Department of Biophysics Physiology and Pathophysiology, Medical University of Warsaw, Chałubińskiego 5, 02-004 Warsaw, Poland; 2 Department of Otolaryngology, Audiology and Phoniatrics, The Children’s Memorial Health Institute, Al. Dzieci Polskich 20, 04-730 Warsaw, Poland; ewelina.sielska@wp.pl; 3 EmergoPharm Sp. z o.o. Sp. K., Jana Ciszewskiego, 02-777 Warsaw, Poland; mc@emergopharm.com (M.C.); artur.wrzosek@emergopharm.com (A.W.); beata.roman@emergopharm.com (B.R.); 4 Faculty of Aviation, Polish Air Force University, Dywizjolony 303 nr 35, 08-521 Dęblin, Poland; j.tomaszewska@law.mil.pl

**Keywords:** otitis externa, lidocaine, glycerol, ear drops, pain, observational study

## Abstract

**Purpose**: The aim of this study was to investigate the safety and efficacy, as well as relief of symptoms after regular use, of 0.5% lidocaine hydrochloride solution in anhydrous glycerol (Auridol) in the form of ear drops, in patients with symptoms of otitis externa (OE) in a real-world setting. **Methods**: This real-world pre–post study included 64 subjects aged 1 to 69 years with symptoms as follow: swelling, pain due to regular exposure to water or caused by frequent use of detergents, pain due to prolonged wearing of earphones, or earwax clogging the external auditory canal. In each subject, following an otoscopic examination and interview given by an ENT, the Auridol treatment was initiated. The product was administered as two drops into the affected ear up to three times daily in patients with symptoms of OE. During each visit, physical and functional symptom were evaluated. In addition, the efficacy of using the product was assessment using a VAS scale. At the end of the study, subjects rated the product according to a Likert scale. **Results**: A statistically significant reduction in perceived pain was observed at *t* = 30′ as well as *t* = 3 days after application. For physical symptoms assessed by an ENT, a statistically significant difference was observed between consecutive scores for two of the assessed parameters (redness and swelling.) The product was rated very highly by the subjects. **Conclusions**: The results suggest that a combination of anhydrous glycerol and 0.5% of lidocaine in the form of ear drops has a positive effect in the treatment of symptoms of OE.

## 1. Introduction

Otitis externa (OE) is defined as inflammation (swelling and redness) of the external ear canal [1]. The condition usually begins with itching, followed by pain, but may also be accompanied by a complete obstruction of the external meatus, and retroauricular swelling. This can result in ears feeling clogged/full ear or hearing impairment. As it often develops after swimming, it is referred to as “swimmer’s ear “or “tropical ear” because repeated exposure to water can make the ear more vulnerable to inflammation. Otitis externa can also develop because of using cotton swabs or earphones, earwax clogging the external auditory canal, or the frequent use of detergents in the external auditory canal, e.g., shampoos, washing gels, or soaps. It is one of most common infection encountered by clinicians [1,2].

About 10% of the population during their lifetime is affected by OE [2,3]. The annual incidence of OE changes with regional variation based of geography (warm and damp climates) and age (among women OE occurs most often occurs around the age of 50, while in men around the age of 70); the incidence is between 1:100 and 1:250 of the general population [4,5,6].

The cause of OE is mostly an infection caused by bacteria. The most common pathogens involved in OE are Pseudomonas aeruginosa and Staphylococcus aureus. Otitis externa can also occur as a polymicrobial infection, viral infection, and rarely as a fungal infection such as Candida or Aspergillus [6,7,8,9].

The method of treatment should be decided after performing differential diagnostics by an ENT (Ear, Nose and Throat specialist). A sudden onset with occurrence of ear pain, otorrhea, itching, and a physical examination revealing an inflammation of the ear canal and pain caused by the pulling the pinna or manipulation of the tragus, are crucial for an appropriate diagnosis. Usually, mild infection can be treated by cleaning the ear canal, use of a topical antiseptic/antimicrobial treatment, and adequate analgesia. In general, OE is responsive to treatment, and usually within 48–72 h. If the infection has spread beyond the ear canal or topical treatment is not possible, antibiotic treatment should be employed [7].

Despite established treatment pathways, pain remains the most distressing symptom of OE and is often inadequately controlled in the early phase of treatment. This is clinically relevant, as pain intensity can significantly impair quality of life and may prompt urgent medical consultation. However, systemic analgesics such as paracetamol and ibuprofen may have a delayed onset of action, with peak plasma concentrations typically occurring 30–60 min after administration, which may not provide sufficiently rapid relief in acute otologic pain.

Recent evidence has therefore focused on locally acting analgesic strategies. Pain relief is a central component of OE management as symptoms can be severe and significantly affect quality of life. Recent evidence highlights the importance of local analgesic strategies. In a 2022 randomized clinical trial in children with acute otitis media, topical 1% lidocaine used as an adjunct to oral analgesics resulted in a significantly greater reduction in patient-reported pain scores compared with oral analgesics alone. Importantly, statistically significant pain reduction was observed as early as 10 min after administration. The authors emphasized that such rapid analgesic effects are unlikely to be explained by paracetamol or ibuprofen alone, given their pharmacokinetic profiles, with peak plasma concentrations typically occurring after 30–60 min. These findings suggest that topical lidocaine may provide a valuable option for achieving rapid pain relief in ear-related conditions [8].

Further supporting this concept, the OPTIMA pragmatic randomized controlled trial evaluated analgesic ear drops as an add-on to standard oral analgesics in children with acute otitis media. Although the study focused on a different clinical condition, it demonstrated the potential value of locally acting, rapid-onset interventions targeting ear pain. Importantly, the results underscore the clinical relevance of addressing pain directly at the site of inflammation and support the broader hypothesis that topical agents may provide meaningful symptomatic benefit in ear canal diseases. Taken together, these findings provide a mechanistic and clinical rationale for investigating lidocaine-based formulations in OE, where direct access to peripheral nerve endings in the external auditory canal may facilitate rapid analgesia [9].

Against this background, the aim of the present study was to evaluate the efficacy and safety of a combination of anhydrous glycerol and 0.5% lidocaine ear drops (Auridol) in patients with symptoms of uncomplicated OE. Additionally, the study assessed patient-reported satisfaction with the product and the speed of symptom relief following regular use.

## 2. Materials and Methods

The study protocol was approved by the Independent Bioethics Committee at the Regional Medical Chamber in Gdansk (Poland) on 8 April 2022.

The study was conducted according to the principles of Good Clinical Practice defined by ICH Topic E6 “Note for Guidance and Good Clinical Practice” (CPMP/ICH/135/95), the Declaration of Helsinki (1964, WMA) and subsequent updates, and the applicable legal regulations A completed STROBE checklist has been included as Supplementary Material Table S1.

### 2.1. Procedure

#### 2.1.1. Participants and Eligibility Criteria

Participants were recruited consecutively from individuals presenting to the Investigating Center with symptoms suggestive of OE. Identification and eligibility assessments were performed by an otolaryngology (ENT) specialist based on otoscopic examination and clinical interview.

Inclusion criteria were based on the presence of symptoms consistent with OE. Following the interview, each subject reported at least one of the following: (1) swelling, pain, or itching of the external auditory canal; (2) pain associated with regular exposure to water or moisture (e.g., swimming); (3) pain related to prolonged use of earphones or cerumen impaction; or (4) pain caused by frequent use of detergents (e.g., shampoos, washing gels, or soaps) in the external auditory canal.

Exclusion criteria included: prior use of any product in the external auditory canal; known acute or chronic dermatological or medical conditions that could affect study outcomes; hypersensitivity to lidocaine, glycerol, or other product components; use of non-steroidal anti-inflammatory drugs within 3 days prior to inclusion; tympanic membrane perforation; recent ear surgery; prolonged symptoms suggestive of bacterial or fungal infection; middle ear inflammation; ear discharge; or participation in another clinical trial.

In addition, patients were excluded if pain or swelling had persisted beyond 3 days from treatment initiation (maximum duration per Instructions for Use), worsened at any time, or remained moderate to severe without clinically meaningful improvement within this period, as assessed by the investigator. These criteria were applied to identify patients requiring alternative management; in such cases, the study product was discontinued at the investigator’s discretion.

Eligible participants who met all criteria were enrolled in the study. Written informed consent was obtained from all participants, or from the parent/legal guardian in the case of children.

#### 2.1.2. Intervention and Study Procedures

Following qualification, treatment with ear drops containing lidocaine hydrochloride (0.5%) in anhydrous glycerol was initiated. The product was administered as two drops into the affected ear up to three times daily according to standardized instructions (head tilted, ear manipulation, and maintenance of position for 5 min to ensure proper distribution).

Clinical assessments were performed three times: baseline (*t* = 0), 30 min after application (*t* = 30 min), and after 3 days of regular use (*t* = 3 days). At each visit, an ENT specialist conducted an otoscopic examination and clinical interview.

### 2.2. Outcome Measures

#### 2.2.1. Clinical Symptom Assessment

Physical signs (redness, swelling, dryness/flaking, cerumen, discharge, and irritation) were assessed by the ENT specialist. Functional symptoms were reported by the subject or, in the case of children, by the parent or legal guardian.

In adults, functional symptoms include burning sensation, fullness, itching, tingling, hearing impairment, and pain during ear manipulation (Table A2). In children under 12 years, symptoms include restlessness, crying, sleep disturbances, and ear pulling or scratching (Table A1).

Symptom severity was graded using a 4-point ordinal scale (0 = none, 1 = mild, 2 = moderate, 3 = severe). This scale reflects routine clinical practice for symptom assessment in observational studies; however, it is not a formally validated disease-specific instrument for OE.

#### 2.2.2. Pain Assessment

Pain intensity was assessed at each visit using the Visual Analog Scale [10], a validated and widely used instrument ranging from 0 (no pain) to 10 (worst imaginable pain). For children, a graphical version of the scale was used.

#### 2.2.3. Patient Satisfaction and Product Evaluation

After 3 days of treatment, participants (or parents/legal guardians) evaluated the product using a Likert scale (1 = not satisfied, 2 = satisfied, 3 = very satisfied, 4 = no opinion) [11]. Assessed domains included ease of application, perceived effectiveness (pain and discomfort reduction), clarity of instructions, and willingness to reuse the product.

Additionally, participants provided an overall product evaluation and indicated their intention to purchase the product.

#### 2.2.4. Safety Assessment

Adverse events and tolerability were monitored throughout the study at each visit.

### 2.3. Statistical Analysis

The statistical analysis was performed using Statistica 13.3 software (StatSoft Poland, Dell Statistica Partner). The normality of quantitative data distribution was assessed using the Shapiro–Wilk test. The quantitative data were summarized using descriptive statistics (mean ± SD, median and range). Categorical variables are expressed as numbers and percentages.

The study sample consisted of 64 patients, which provided at least 81% statistical power to detect differences of at least 20% between groups at a significance level of α = 0.05.

Differences in product evaluation over time (baseline: *t* = 0′, 30 min after application: *t* = 30′, and after 3 days of regular use: *t* = 3 days) were analyzed according to data type and distribution. For categorical variables, the chi-square test or Fisher’s exact test was applied, as appropriate. For repeated quantitative measurements, the non-parametric Friedman ANOVA test was used because of the study design and non-normal distribution of data.

In the case of statistically significant overall differences across time points, post hoc pairwise comparisons were performed between *t* = 0′ vs. *t* = 30′, and *t* = 0′ vs. *t* = 3 days. To control multiple comparisons, a Bonferroni correction was applied, with the adjusted significance level set at *α* = 0.025. A two-sided *p*-value < 0.05 was considered statistically significant unless otherwise specified.

## 3. Results

### 3.1. Participants

**Figure 1:**
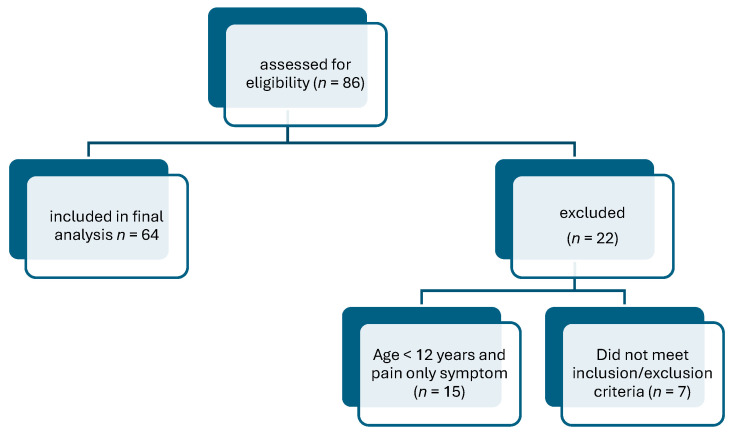
Flowchart of patient selection.

This was a prospective, open-label, single-center clinical study. Participants were recruited on a voluntary basis to the Investigating Centre from May 2022 to August 2022. Initially 86 subjects were considered for the study group. However, 22 participants were excluded. Seven patients did not meet inclusion or exclusion criteria, and 15 participants under the age of 12 years old were excluded because the sole symptom of pain was not sufficient to suggest OE. Finally, the analysis presented in this article was based on a group of participants who, apart from the symptoms of pain, had additional redness or swelling confirmed by the ENT. Conclusively, 64 subjects (47 females and 17 males) were included in the study (Figure 1). The average age of the group was 34 ± 20 years, median 36 years, and range 1–69 years. The mean age in the group under 12 years was 7.7 ± 2.9 years, with a median of 8.0 years and an age range of 1–11 years. The mean age in the group over 12 years was 41.9 ± 16.0 years, with a median of 43.5 years and an age range of 12–69 years. Baseline (D0) clinical characteristics of the study population, including symptom profiles and age distribution by study group, are presented in Table 1 and Table 3. All participants or parent/legal guardian of a child subject included in the study were required to submit written consent prior to participating in the study.

### 3.2. ENT Assessment

With regard to the physical symptoms assessed by the ENT, a statistically significant difference was found between consecutive scores for two assessed parameters: redness and swelling. Here, the obtained *p*-value was *p* < 0.001 and *p* = 0.005 respectively. To assess whether there were statistically significant differences between *t* = 0′, *t* = 30′, and *t* = 3 days, the non-parametric Wilcoxon paired test was used followed by the Bonferroni correction because of multiple comparisons (*α* = 0.025). At the inclusion visit on day 0, the severity of redness was reduced after 30 min, but there was no statistically significant difference, while swelling was nearly the same level. A statistically significant reduction in redness as well as swelling was observed after 3 days of application of the product. After 3 days, the number of subjects with no redness increased from 46.9% (at the initial assessment) to 87.5%, while the number of subjects with no swelling increased from 79.7% (at the initial assessment) to 100%. For other assessed physical symptoms, an increase in the number of subjects in whom the ENT confirmed an absence of dryness/flaking skin in the ear canal, earwax, or other symptoms (e.g., pain when pulling the ear/ tenderness when moving) was also observed; however, the difference was not statistically significant. After 3 days, the ENT confirmed that none of the subjects included in the study had severe or moderate dryness/flaking skin in the ear canal, and mild dryness/flaking skin in the ear canal was observed in 3.1% of the group. Moreover, the group of participants who reported severe earwax was reduced from 7.8% to 3.1%, and the number of subjects who reported no earwax increased from 39 (60.9%) to 51 (79.7%) (Table A3 and Table A4).

In the adult subjects, with regards to the functional symptoms, a statistically significant difference was found for two of the six assessed symptoms (sensation of congestion/fullness and itching); here the *p*-values were 0.007 and *p* < 0.001, respectively. For the sensation of warm/burning, the obtained *p*-value was at the border of the significance level. For the sensation of congestion/fullness and itching, a statistically significant difference was found after 3 days of application of the product. After 3 days, the number of subjects with no sensation of congestion/fullness increased from 80% to 100%. However, a statistically significant increase was observed in the number of subjects who reported a reduction in itching 30 min after application of the product (from 45% to 65%), and 3 days after application of the product (from 45% to 98%). For the other functional symptoms, there was also an increase in the number of subjects who did not report them, but this was not statistically significant. After 3 days of regular use of the product none of the subjects reported a warm/burning sensation or stinging/pricking, and 2% of subjects reported a feeling of hearing loss, while 98% did not (Table 2).

In the group of subjects under 12 years old, there was an increase in the number of subjects in whom the ENT confirmed a reduction of assessed symptoms. After 3 days of using the product, scratching/pulling was observed in none of the participants. Similarly there was no pain observed when pulling the ear/tenderness when moving the ear or jaw (Table 3 and Table A5).

**Table 3:** Severity of scratching/pulling and related symptoms in children (<12 years) over time.

	Scratching/Pulling				Others (Pain When Pulling the Ear/Tenderness When Moving the Ear or Jaw)			
The degree of severity	*t* = 0	*t* = 30′	*t* = 3 days	*p*	*t* = 0	*t* = 30′	*t* = 3 days	*p*
None	73.3% (11)	93.3% (14)	100% (15)	0.105	66.7% (10)	93.3% (14)	100% (15)	0.073
Mild	6.7% (1)	6.7% (1)	0% (0)		20% (3)	6.7% (1)	0% (0)	
Moderate	20% (3)	(0)	0% (0)		13.3% (2)	0	0% (0)	

### 3.3. VAS Scale

**Figure 2:**
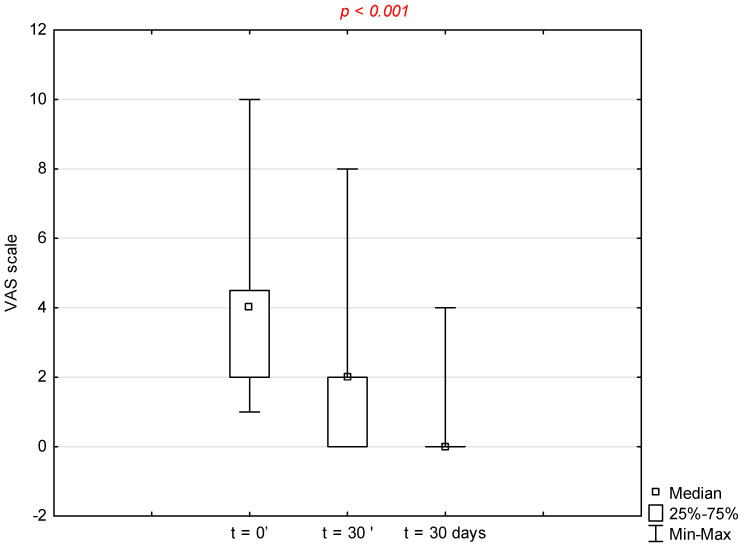
Distribution of Visual Analog Scale (VAS) scores over time (D0, 30 min post-intervention, and Day 3)—evaluation of the declared product properties.

A statistically significant difference was observed between *t* = 0′, *t* = 30′, and *t* = 3 days (*p* < 0.001) when assessing the level of pain using a VAS scale. To assess whether there were statistically significant differences between *t* = 0′, *t* = 30′, and *t* = 3 days, a non-parametric Wilcoxon paired test was used followed by the Bonferroni correction because of multiple comparisons (*α* = 0.025). A statistically significant reduction in perceived pain was observed at *t* = 30′ as well as at *t* = 3 days after application of the product (Figure 2). The median and the range of the VAS scale was 4 (1–10), 2 (0–8), and 0 (0–4) respectively for the first, second, and third assessment (Table A6).

The product was rated very highly by study participants based on the questionnaire evaluation. Ninety-eight percent of subjects reported being very satisfied or satisfied with the reduction of ear discomfort, and 98% reported satisfaction with pain reduction. These results are consistent with the findings confirming the product’s effectiveness in reducing pain based on the VAS score analysis. Moreover, 90% of participants reported that the product acted quickly, with an onset of effect after approximately 5 min, and 100% of subjects experienced relief after the first application.

## 4. Discussion

Otitis externa is a common clinical condition. Milder cases are usually treated in primary care, while severe OE accounts for a significant proportion of emergency presentations to ENTs. Topical antibiotics are widely used for first-line treatment. Johsoton et al. [12] showed that the addition of a steroid and antibiotic to an antibacterial agent resulted in better control of OE symptoms. Otitis externa is also managed by cleaning the ear canal and the dosing of topical medications in the form of spray or drops [13]. Jinn et al. [14] showed that in the presence of a tympanic membrane perforation, combined ear drop preparations may have an ototoxic effect.

Auridol is a combination of anhydrous glycerol and a 0.5% solution of lidocaine in the form of ear drops. Glycerol is a transparent hydroscopic liquid that achieves its main effect through its ability to bind water. Due to this property, glycerol applied in situ helps to remove excess water from swollen tissue (osmotic effect), thus reducing swelling and pain intensity [15,16]. Lidocaine used ancillary as a local anesthetic, intensifies this effect and additionally relieves ear pain [17]. The use of local anesthetics in acute OE, was recommended in guidelines in 2006 and then subsequently established as standard practice [17]. In uncomplicated ear infection and earache, lidocaine is the local anesthetic of choice for the topical treatment of pain [18]. This makes it possible to achieve the desired local anesthetic effect while avoiding side effects such as its high potential for addiction. Bolt et al. showed that lidocaine solution applied for the topical treatment of ear pain in acute otitis media works fast (the maximum effect is achieved within 2–5 min), is effective for 30–45 min and has a high level of bioavailability [19].

Given that uncomplicated ear infections are common in childhood, the use of alternative treatments, such as non-antibiotic ear drops or local anesthetics prior to initiating antibiotic therapy, may reduce the development of antibiotic resistance and minimize the risk of adverse effects [20]. Similarly, OE can occur among swimmers and the scuba divers, because swimming and diving have become popular social recreational activities. As suggested by Maclean et al. the high prevalence of sulphonamide-resistant genes in the environment may lead to the development of resistance to sulphonamides, which will lead to spread of infection and affect treatment plans [21].

Previous studies have evaluated the efficacy of non-antibiotic and antibiotic therapies for OE in the absence of otitis media. In a study by Tsikoudas et al., no statistically significant differences were observed in patient and physician assessments after 11 days of treatment in either group, indicating that the treatment method did not significantly affect the outcome. However, the authors noted that patient compliance with self-treatment was poor, although similar between the two groups. Additionally, the diagnosis in both groups was not confirmed by microbiological smear, and the study was limited by its small sample size.

In 2014, the guideline recommendations for managing acute otitis externa (AOE) were updated by Rosenfeld et al. [17] and replaced the guideline published in 2006 by the American Academy of Otolaryngology–Head and Neck Surgery Foundation [22]. Based on clinical trials and systematic reviews, Rosenfeld et al. noticed that about one-third of AOE cases are treated with oral antibiotics, which are ineffective. According to their guideline recommendations, treatment for AOE should include analgesics, and ear drops containing an antiseptic, antibiotics, and corticosteroids alone, or in combination, highlighting that ear drops are very effective because of the high local concentrations of drug in the ear canal. Moreover, in 2021 by Smith et al. [23] provided a consensus definition for the diagnostic criteria for AOE. Topical analgesic ear drops were also listed as a treatment option for otalgia associated with AOM in guidelines from the American Academy of Pediatrics. However, the supporting evidence for this recommendation remains limited, particularly in pediatric populations, as experimental randomized studies in children are scarce [6].

Early and adequate pain management during the initial stages of ear infections, including AOM, is crucial. If pain is present, it should be addressed, particularly within the first 24 h of an episode. Effective pain management in AOM requires a thorough assessment of pain regardless of the decision to prescribe antibiotics, as antibiotics do not typically alleviate ear pain within the first 24 h and have a limited impact on pain relief even after three to seven days.

The aim of this study was to assess the efficacy and safety of ear drops containing a 0.5% lidocaine hydrochloride solution in anhydrous glycerol in a real-world pre–post setting. We considered a group of 64 subjects, age range 1 to 69 years, who used 2 drops up to three times a day for 3 days in the ears diagnosed with symptoms of OE (children under the supervision of adults or a doctor). We observed that the diagnosed physical symptoms after 3 days of ear drop application were significantly reduced. In 87.5% and 12.5% of participants, respectively, no redness was observed, or it was assessed as mild. Swelling had completely resolved, and earwax was assessed as still severe in only two subjects (3.1%).

Moreover, among the functional symptoms, itching was also significantly reduced in most of the participants who reported it initially. None of the participants reported feeling clogged ears after regular use of the product. In addition, pain relief is an essential part of the treatment OE (the highly sensitive periosteum of the bony ear canal is usually involved in the inflammatory process). Based on the VAS scale assessed severity of pain, we noticed a statistically significant reduction of pain after 30 min of application, as well as after 3 days of regular application.

In addition, during each clinical visit, an otolaryngology specialist evaluated the product’s performance and monitored for any adverse events. Importantly, none of the subjects included in the study exhibited allergic reactions or other side effects during product use, and no medical incidents were reported throughout the study period.

Our findings indicate that ear drops containing 0.5% lidocaine in anhydrous glycerol are effective and exhibit a favorable safety profile for the management of uncomplicated ear infections. In the context of the existing literature, the observed improvements in pain and clinical symptoms are consistent with evidence supporting the effectiveness of topical therapies in OE. Systematic reviews, including a Cochrane review by Vikram Kaushik et al. [24] indicate that a wide range of topical agents provide clinical benefit, although no single intervention has demonstrated clear superiority. Similarly, current recommendations from the American Academy of Otolaryngology–Head and Neck Surgery Foundation [17] and the evidence-based emergency care review by Hershil Khatri et al. [25], emphasize topical treatment as a first-line therapy, but do not identify a preferred formulation. In this context, the magnitude and rapid onset of pain relief observed in our study appears comparable to previously reported outcomes for locally acting analgesic strategies, including topical lidocaine preparations described by David Bolt et al. [19]. However, due to differences in study design, patient populations, and outcome measures, such comparisons should be interpreted with caution and no conclusions regarding relative efficacy can be drawn. In Europe, lidocaine in anhydrous glycerol ear drops is available in concentrations ranging from 0.5% to 2.0%. Ahmed et al. noted that the clinical efficacy of glycerin-ichthammol in OE may be due to the anti-inflammatory action of ichthammol or an osmotic effect of glycerin on the oedematous ear canal [26]. In addition, Mather et al., based on their observations, highlight the inappropriate use of oral antibiotics and recommends their substitution with ototopical drops [27]. According to Mösges et al. [18] neomycin sulfate and gramicidin (PS) applied in patients with acute bacterial OE, show results superior to glycerol. However, it should be noted that our study only investigated the efficacy and safety ear drops containing 0.5% lidocaine hydrochloride solution in anhydrous glycerol in subjects with initial symptoms of OE; patients with prolonged pain or swelling caused by inflammation of a bacteria were not considered. These ear drops can be used in patients with a wax plug occluding the external auditory canal, in which the patient (due to external factors, e.g., the improper use of cotton buds) has pushed the wax medially or scratched themself, causing pain.

Moreover, the product was rated very highly by the study subjects based on the questionnaire evaluation findings. The high levels of patient-reported satisfaction observed in this study further support the clinical effectiveness of the investigated product. Nearly all of the participants reported being satisfied with the reduction of both ear discomfort and pain, which is consistent with the significant improvement observed in the VAS scores. Importantly, the rapid onset of action, reported by 90% of subjects within approximately 5 min, and complete relief after the first application in all participants, may represent a key factor contributing to overall treatment satisfaction. These findings suggest that beyond objective symptom reduction, the product provides a fast and perceptible clinical benefit, which is particularly relevant in acute conditions where immediate relief is highly valued. Such rapid symptomatic improvement may also enhance treatment adherence and overall patient experience, especially in pediatric populations where discomfort-related distress is more pronounced.

A key limitation of this study is the absence of a direct or indirect comparison with other topical or systemic treatments commonly used in OE. As this was a single-arm, real-world pre–post observational study, no comparator group (placebo or active treatment) was included. Consequently, although significant improvements in pain and clinical symptoms were observed, these findings cannot be interpreted in terms of relative efficacy. It is therefore not possible to determine whether the observed effects are superior, equivalent, or inferior to standard therapies such as topical antibiotics, antiseptics, corticosteroid-containing preparations, or systemic analgesics. Furthermore, part of the observed improvement may reflect the natural, often self-limiting course of uncomplicated OE. Although our findings have been contextualized with previously published studies, such cross-study comparisons should be interpreted with caution due to differences in study design, patient populations, and outcome measures. Future randomized controlled trials with appropriate comparator arms are warranted to establish comparative effectiveness and to better define the role of this formulation in clinical practice.

Another limitation concerns outcome assessment. Symptom severity was evaluated using a 4-point ordinal scale based on clinical evaluation and patient-reported outcomes. Although this reflects routine clinical practice, it is not a validated disease-specific instrument, which may have introduced measurement bias and limit comparability with other studies. Pain assessment was strengthened using the VAS scale; however, other symptom domains were assessed using non-validated scales.

Several potential sources of bias should also be acknowledged. First, the lack of blinding may have introduced both performance and detection bias, as knowledge of treatment could have influenced both the patient-reported outcomes and clinician assessments. Second, the absence of a control group increases the risk of confounding, particularly related to the natural resolution of ear infection, concurrent treatments, or variations in disease severity at baseline. Third, selection bias may be present due to the single-center design and the relatively homogeneous study population. These factors may have led to an overestimation of the observed treatment effect and limit the internal validity of the findings.

Furthermore, the relatively small sample size and single-center design may limit the robustness and generalizability of the results. The limited number of participants reduces statistical precision and may increase the risk of type II error, while also restricting more detailed subgroup analyses. The short follow-up period (3 days) further limits conclusions regarding long-term efficacy, durability of response, and recurrence rates.

It is also noteworthy that a subset of patients (7/64; 10.9%) did not demonstrate meaningful improvement in redness or pain and required further outpatient management. In these cases, it remains unclear whether the lack of response was related to insufficient effect of the intervention, the need for additional procedures such as ear canal cleaning, or progression of infection. This highlights the heterogeneity of clinical response and the need for further stratification in future studies.

Finally, although no adverse events were reported, the short observation period limits the ability to fully assess the safety profile of the intervention over time. In addition, two physicians were involved in the study. However, each assessed separate groups of patients, which precluded evaluation of interobserver reliability (e.g., kappa statistic), and this should be considered when interpreting the consistency of clinical assessments.

## 5. Conclusions

The findings indicate that a formulation combining anhydrous glycerol with 0.5% lidocaine, administered as otic drops, is effective in alleviating early symptoms of otitis externa and may be considered a first-line therapeutic option for uncomplicated cases. In addition, this formulation may be suitable for use in patients with external auditory canal obstruction due to cerumen impaction, particularly when external factors such as improper cotton swab use or self-induced abrasions have resulted in medial displacement of cerumen or the development of pain and discomfort.

## Figures and Tables

**Table 1:** Changes in the severity of physical ear symptoms (redness, swelling, dryness, earwax, and others) at baseline, 30 min, and Day 3.

	Physical Symptoms % (*n*)							
	Redness				Swelling			
The degree of severity	D0	po T30′	D3	*p*	D0	po T30′	D3	*p*
None	46.9% (30)	53.1% (34)	87.5% (56)	<0.001	79.7% (51)	87.5% (56)	100% (64)	0.005
Mild	48.4% (31)	45.3% (29)	12.5% (8)		18.7% (12)	12.5% (8)	0% (0)	
Moderate	4.7% (3)	1.6% (1)	0% (0)		1.6% (1)	0% (0)	0% (0)	
Severe	0% (0)	0% (0)	0% (0)		0% (0)	0% (0)	0% (0)	
	Dryness/ flaking skin in the ear canal				Earwax			
The degree of severity	D0	po T30′	D3	*p*	D0	po T30′	D3	*p*
None	87.5% (56)	92.2% (59)	96.9% (62)	0.099	60.9% (39)	64.1% (41)	79.7% (51)	0.384
Mild	7.8% (5)	7.8% (5)	3.1% (2)		20.4% (13)	18.7% (12)	9.4% (6)	
Moderate	4.7% (3)	0% (0)	0% (0)		10.9% (7)	10.9% (7)	7.8% (5)	
Severe	0% (0)	0% (0)	0% (0)		7.8% (5)	6.3% (4)	3.1% (2)	
	Secretion leakage				Others			
The degree of severity	D0	po T30′	D3	*p*	D0	po T30′	D3	*p*
None	100% (64)	100% (64)	100% (64)	1	89% (57)	96.9% (62)	98.4% (63)	0.241
Mild	0.0% (0)	0.0% (0)	0.0% (0)		9.4% (6)	3.1% (2)	1.6% (1)	
Moderate	0.0% (0)	0.0% (0)	0.0% (0)		1.6% (1)	0.0% (0)	0.0% (0)	
Severe	0.0% (0)	0.0% (0)	0.0% (0)		0.0% (0)	0.0% (0)	0.0% (0)	

**Table 2:** Distribution and temporal changes in the severity of functional ear symptoms in adults (D0, post T, and D3).

	Adult Functional Symptoms % (*n*)							
	Warm/burning sensation				Feeling clogged/full			
The degree of severity	D0	po T30′	D3	*p*	D0	po T30′	D3	*p*
None	81.7% (40)	85.8% (42)	100.0% (49)	0.050	79.6% (39)	81.6% (40)	100.0% (49)	0.007
Mild	16.3% (8)	12.2% (6)	0		16.3% (8)	18.4% (9)	0.0% 0	
Moderate	2.0% (1)	2.0% (1)	0.0% 0		4.1% (2)	0.0% 0	0.0% 0	
Severe	0.0% 0	0.0% 0	0.0% 0		0.0% 0	0.0% 0	0.0% 0	
	Itching				Stinging/prickling			
The degree of severity	D0	po T30′	D3	*p*	D0	po T30′	D3	
None	44.9% (22)	65.3% (32)	98% (48)	<0.001	93.9% (46)	95.9% (47)	100.0% (49)	0.234
Mild	20.4% (10)	26.6% (13)	2.0% (1)		6.1% (3)	4.1% (2)	0.0% 0	
Moderate	32.7% (16)	6.1% (3)	0.0% 0		0.0% 0	0.0% 0	0.0% 0	
Severe	2.0 (1)	2.0 (1)	0.0% 0		0.0% 0	0.0% 0	0.0% 0	
	Feeling of hearing loss				Others			
The degree of severity	D0	after 30′	D3	*p*	D0	po T30′	D3	*p*
None	91.8% (45)	89.8% (44)	98% (48)	0.203	85.7% (42)	87.8% (43)	98% (48)	0.176
Mild	8.2% (4)	6.1% (3)	2.0% (1)		12.3% (6)	12.2% (6)	2.0% (1)	
Moderate	0% (0)	4.1% (2)	0.0% (0)		2.0% (1)	0.0% 0	0.0% 0	

## Data Availability

The data presented in this study are available on request from the corresponding author.

## Supplementary Materials

The following supporting information can be downloaded at: https://www.mdpi.com/article/10.3390/clinpract16050090/s1, Table S1: STROBE Statement—checklist of items that should be included in reports of observational studies.

## Appendix A

**Table A1:** Physical and Functional Symptom Severity in Children Under 12 Years (0–3 Scale).

**Children Under 12 Years Old**		**None** **(0)**	**Mild** **(1)**	**Moderate** **(2)**	**Severe** **(3)**
**Physical signs**	Redness				
	Edema/Swelling				
	Dryness/flaking skin in the ear canal				
	Earwax				
	Secretion leak				
	Others, such as irritation				
If possible the subject reported the functional signs:					
		**None** **(0)**	**Mild** **(1)**	**Moderate** **(2)**	**Severe** **(3)**
**Functional signs**	Anxiety				
	Cry				
	Trouble sleeping				
	Ear scratching/ear pulling				
	Others				

**Table A2:** Physical and Functional Symptom Severity in Adults/Children over 12 years of age (0–3 Scale).

**Adults/Children over 12 Years of Age**		**None** **(0)**	**Mild** **(1)**	**Moderate** **(2)**	**Severe** **(3)**
**Physical signs**	Redness				
	Edema/Swelling				
	Dryness/flaking skin in the ear canal				
	Earwax				
	Secretion leak				
	Others, such as irritation				
If possible the subject reported the functional signs:					
		**None** **(0)**	**Mild** **(1)**	**Moderate** **(2)**	**Severe** **(3)**
**Functional signs**	Warm/burning sensation				
	Clogging/fullness sensation				
	Itching				
	Stinging/tingling sensation				
	Feeling of being hard of hearing				
	Others, e.g., pain when pulling the ear/tenderness when moving the ear or jaw				

**Table A3:** The severity of physical ear symptoms at baseline.

	Adults’ Physical Symptoms D0% (*n*)					
The Degree of Severity	Redness	Swelling	Dryness/Flaking Skin in the Ear Canal	Earwax	Secretion Leakage	Others
none	46.9% (30)	79.7% (51)	87.5% (56)	60.9% (39)	100% (64)	89% (57)
mild	48.4% (31)	18.70% (12)	7.8% (5)	20.4% (13)	0.0% (0)	9.4% (6)
moderate	4.7% (3)	1.6% (1)	4.7% (3)	10.9% (7)	0.0% (0)	1.6% (1)
severe	0.0% (0)	0.0% (0)	0.0% (0)	7.8% (5)	0.0% (0)	0.0% (0)

**Table A4:** The severity of adults’ functional symptoms at baseline.

	Adults’ Functional Symptoms D0 % (*n*)					
The Degree of Severity	Warm/Burning Sensation	Feeling Clogged/Full	Itching	Stinging/ Prickling	Feeling of Hearing Loss	Others
none	81.7% (40)	79.6% (39)	44.9% (22)	93.9% (46)	91.8% (45)	85.7% (42)
mild	16.3% (8)	16.3% (8)	20.4% (10)	6.1% (3)	8.2% (4)	12.3% (6)
moderate	2.0% (1)	4.1% (2)	32.7% (16)	0.0% (0)	0.0% (0)	2.0% (1)
severe	0.0% (0)	0.0% (0)	2.0% (1)	0.0% (0)	0.0% (0)	0.0% (0)

**Table A5:** The severity of functional symptoms in children (<12 years) at baseline.

	Children Under 12 Years Old Functional Signs D0 % (*n*)	
The Degree of Severity	Scratching/ Pulling	Others (Pain When Pulling the Ear/Tenderness When Moving the Ear or Jaw)
none	73.3% (11)	66.7% (10)
mild	6.7% (1)	2% (3)
moderate	20% (3)	13.3% (2)

**Table A6:** Summary of Baseline VAS Scores (Median, Min–Max, Q1–Q3).

	VAS at Baseline		
	All Subjects	Adults/Children over 12 Years of Age	Children (<12 Years)
Median (Min–Max)	4 (1–10)	4 (1–10)	3 (1–6)
Q1–Q3	2.0–4.5	2–5	1–4

## References

[1] Long M. (2013). Otitis externa. Pediatr. Rev..

[2] Russell J.D., Donnelly M., McShane D.P., Alun-Jones T., Walsh M. (1993). What causes acute otitis externa?. J. Laryngol. Otol..

[3] Beers S.L., Abramo T.J. (2004). Otitis externa review. Pediatr. Emerg. Care.

[4] Neher A., Nagl M., Scholtz A. (2008). Otitis externa. Hno.

[5] Hoadley A.W., Knight D.E. (1975). External otitis among swimmers and nonswimmers. Arch. Environ. Health.

[6] Villedieu A., Papesh E., Weinberg S.E., Teare L., Radhakrishnan J., Elamin W.F. (2018). Seasonal variation of Pseudomonas aeruginosa in culture positive otitis externa in South East England. Epidemiol. Infect..

[7] Wiegand S., Berner R., Schneider A., Lundershausen E., Dietz A. (2019). Otitis Externa. Dtsch. Arztebl. Int..

[8] Kara A., Büyükcam A., Sütcü M., Sali E., Bozdemir Ş.E., Kara M., İlarslan E.Ç., Kaya C., Karakaşlılar S., Sönmez G. (2022). The effectiveness of topical 1% lidocaine with systemic oral analgesics for ear pain with acute otitis media. Int. J. Pediatr. Otorhinolaryngol..

[9] de Sévaux J.L.H., Damoiseaux R.A.M.J., Hullegie S., Sanders E.A.M., de Wit G.A., Zuithoff N.P.A., Yardley L., Anthierens S., Little P., Hay A.D. (2023). Effectiveness of analgesic ear drops as add-on treatment to oral analgesics in children with acute otitis media: Study protocol of the OPTIMA pragmatic randomised controlled trial. BMJ Open.

[10] Hawker G.A., Mian S., Kendzerska T., French M. (2011). Measures of adult pain: Visual Analog Scale for Pain (VAS Pain), Numeric Rating Scale for Pain (NRS Pain), McGill Pain Questionnaire (MPQ), Short-Form McGill Pain Questionnaire (SF-MPQ), Chronic Pain Grade Scale (CPGS), Short Form-36 Bodily Pain Scale (SF-36 BPS), and Measure of Intermittent and Constant Osteoarthritis Pain (ICOAP). Arthritis Care Res..

[11] Streiner D.L., Norman G.R., Cairney J. (2014). Norman, and John Cairney, Health Measurement Scales: A Practical Guide to Their Development and Use.

[12] Johnston M.N., Flook E.P., Mehta D., Mortimore S. (2006). Prospective randomised single-blind controlled trial of glacial acetic acid versus glacial acetic acid, neomycin sulphate and dexamethasone spray in otitis externa and infected mastoid cavities. Clin. Otolaryngol..

[13] Ruddy J., Bickerton R.C. (1992). Optimum management of the discharging ear. Drugs.

[14] Jinn T.H., Kim P.D., Russell P.T., Church C.A., John E.O., Jung T.T. (2001). Determination of ototoxicity of common otic drops using isolated cochlear outer hair cells. Laryngoscope.

[15] Hornigold R., Gillett D., Kiverniti E., Harries M. (2008). The management of otitis externa: A randomised controlled trial of glycerol and icthammol ribbon gauze versus topical antibiotic and steroid drops. Eur. Arch. Otorhinolaryngol..

[16] Nilssen E., Wormald P.J., Oliver S. (1996). Glycerol and ichthammol: Medicinal solution or mythical potion?. J. Laryngol. Otol..

[17] Rosenfeld R.M., Schwartz S.R., Cannon C.R., Roland P.S., Simon G.R., Kumar K.A., Huang W.W., Haskell H.W., Robertson P.J. (2014). Clinical practice guideline: Acute otitis externa. Otolaryngol. Head Neck Surg..

[18] Mösges R., Kaatz V., Schmalz P., Meiser P., Eschmann K. (2010). Glycerol lidocaine eardrops for the treatment of acute abacterial otitis externa. Arzneimittelforschung.

[19] Bolt P., Barnett P., Babl F.E., Sharwood L.E. (2008). Topical Lignocaine for Pain Relief in Acute Otitis Media: Results of a Double-Blind Placebo-Controlled Randomised Trial. Arch. Dis. Child..

[20] Gruber M., Damry D., Ibrahim N., Glikman D., Ronen O. (2021). Pediatric acute otitis externa: Characteristics and predictors for hospital admission. Int. J. Pediatr. Otorhinolaryngol..

[21] Maclean K., Njamo F.O.J.P., Serepa-Dlamini M.H., Kondiah K., Green E. (2022). Antimicrobial Susceptibility Profiles among Pseudomonas aeruginosa Isolated from Professional SCUBA Divers with Otitis Externa, Swimming Pools and the Ocean at a Diving Operation in South Africa. Pathogens.

[22] Rosenfeld R.M., Brown L., Cannon C.R., Dolor R.J., Ganiats T.G., Hannley M., Kokemueller P., Marcy S.M., Roland P.S., Shiffman R.N. (2006). Clinical practice guideline: Acute otitis externa. Otolaryngol. Head Neck Surg..

[23] Smith M.E., Hardman J.C., Mehta N., Jomes G.H., Mandavia R., Anderson C., Khan M., Abdelaziz A., Al-Dulaimy B., Amin N. (2021). Acute otitis externa: Consensus definition, diagnostic criteria and core outcome set development. PLoS ONE.

[24] Kaushik V., Malik T., Saeed S.R. (2011). Cochrane review: Interventions for acute otitis externa. Evid.-Based Child Health.

[25] Khatri H., Huang J., Guazzo E., Bond C. (2021). Review article: Topical antibiotic treatments for acute otitis externa: Emergency care guidelines from an ear, nose and throat perspective. Emerg. Med. Australas. EMA.

[26] Ahmed K., Roberts M.L., Mannion P.T. (1995). Antimicrobial activity of glycerine-ichthammol in otitis externa. Clin. Otolaryngol. Allied Sci..

[27] Mather M.W., Mohammed H., Wilson J.A. (2022). Improving patient care pathways in otitis externa. Fam. Pract..

