# Supplementary material for: Efficacy and Safety of Glycerol Lidocaine Ear Drops in the Non-Antibiotic Treatment of Otitis Externa Symptoms—An Observational Study
Source: Clin Pract. 2026 Apr 30;16(5):90. doi: 10.3390/clinpract16050090 (PMC13206003; doi:10.3390/clinpract16050090)
Supplement: Supplementary file 1 [file clinpract-16-00090-s001.zip › clinpract-4218856-supplementary.pdf]

Table S1. STROBE Statement—checklist of items that should be included in reports of observational studies

	Item No	Recommendation
**Title and abstract**	1	(*a*) Indicate the study's design with a commonly used term in the title or the abstract **(page 1)**
		(*b*) Provide in the abstract an informative and balanced summary of what was done and what was found (**page 1)**
**Introduction**		
Background/rationale	2	Explain the scientific background and rationale for the investigation being reported **(page 1)**
Objectives	3	State specific objectives, including any prespecified hypotheses **(page 1)**
**Methods**		
Study design	4	Present key elements of study design early in the paper
Setting	5	Describe the setting, locations, and relevant dates, including periods of recruitment, exposure, follow-up, and data collection **pages 3,4 and 5**
Participants	6	**Pages 3, 4 and 5**
Variables	7	Clearly define all outcomes, exposures, predictors, potential confounders, and effect modifiers. Give diagnostic criteria, if applicable **(pages 6 and 7)**
Data sources/ measurement	8*	For each variable of interest, give sources of data and details of methods of assessment (measurement). **(pages 4 and 5)**
Bias	9	Describe any efforts to address potential sources of bias **(page 11 and 12)**
Study size	10	Explain how the study size was arrived at **(page 5)**
Quantitative variables	11	Explain how quantitative variables were handled in the analyses. If applicable, describe which groupings were chosen and why (**page 5)**
Statistical methods	12	(*a*) Describe all statistical methods, including those used to control for confounding **(page 5)**

Continued on next page

**Results**		
Participants	13*	(a) Report numbers of individuals at each stage of study—eg numbers potentially eligible, examined for eligibility, confirmed eligible, included in the study, completing follow-up, and analysed **(page 5 and 6)**
		(c) Consider use of a flow diagram **(page 6)**
Descriptive data	14*	(a) Give characteristics of study participants (eg demographic, clinical, social) and information on exposures and potential confounders **(page 5)**
**Discussion**		
Key results	18	Summarise key results with reference to study objectives **(pages 12 and 13)**
Limitations	19	Discuss limitations of the study, taking into account sources of potential bias or imprecision. Discuss both direction and magnitude of any potential bias **(pages 12 and 13)**
Interpretation	20	Give a cautious overall interpretation of results considering objectives, limitations, multiplicity of analyses, results from similar studies, and other relevant evidence **(page 13)**
Generalisability	21	Discuss the generalisability (external validity) of the study results **(page 13)**
**Other information**		
Funding	22	Give the source of funding and the role of the funders for the present study and, if applicable, for the original study on which the present article is based **(page 14)**
